# Platelet-Derived PCSK9 Is Associated with LDL Metabolism and Modulates Atherothrombotic Mechanisms in Coronary Artery Disease

**DOI:** 10.3390/ijms222011179

**Published:** 2021-10-16

**Authors:** Álvaro Petersen-Uribe, Marcel Kremser, Anne-Katrin Rohlfing, Tatsiana Castor, Kyra Kolb, Valerie Dicenta, Frederic Emschermann, Bo Li, Oliver Borst, Dominik Rath, Karin Anne Lydia Müller, Meinrad Paul Gawaz

**Affiliations:** Department of Cardiology and Angiology, University Hospital Tübingen, Eberhard Karls University Tübingen, 72076 Tübingen, Germany; Alvaro.Petersen@med.uni-tuebingen.de (Á.P.-U.); Marcel.Kremser@med.uni-tuebingen.de (M.K.); Anne-Katrin.Rohlfing@med.uni-tuebingen.de (A.-K.R.); Tatsiana.Castor@med.uni-tuebingen.de (T.C.); Kyra.Kolb@med.uni-tuebingen.de (K.K.); Valerie.Dicenta@med.uni-tuebingen.de (V.D.); Frederic.Emschermann@med.uni-tuebingen.de (F.E.); Bo.Li@med.uni-tuebingen.de (B.L.); Oliver.Borst@med.uni-tuebingen.de (O.B.); Dominik.Rath@med.uni-tuebingen.de (D.R.); K.Mueller@med.uni-tuebingen.de (K.A.L.M.)

**Keywords:** PCSK9, platelets, atherothrombosis, thrombo-inflammation, LDL

## Abstract

Platelets play a significant role in atherothrombosis. Proprotein convertase subtilisin/kexin type 9 (PCSK9) is critically involved in the regulation of LDL metabolism and interacts with platelet function. The effect of PCSK9 in platelet function is poorly understood. The authors of this article sought to characterize platelets as a major source of PCSK9 and PCSK9’s role in atherothrombosis. In a large cohort of patients with coronary artery disease (CAD), platelet count, platelet reactivity, and platelet-derived PCSK9 release were analyzed. The role of platelet PCSK9 on platelet and monocyte function was investigated in vitro. Platelet count and hyper-reactivity correlated with plasma LDL in CAD. The circulating platelets express on their surface and release substantial amounts of PCSK9. Release of PCSK9 augmented platelet-dependent thrombosis, monocyte migration, and differentiation into macrophages/foam cells. Platelets and PCSK9 accumulated in tissue derived from atherosclerotic carotid arteries in areas of macrophages. PCSK9 inhibition reduced platelet activation and platelet-dependent thrombo-inflammation. The authors identified platelets as a source of PCSK9 in CAD, which may have an impact on LDL metabolism. Furthermore, platelet-derived PCSK9 contributes to atherothrombosis, and inhibition of PCSK9 attenuates thrombo-inflammation, which may contribute to the reported beneficial clinical effects.

## 1. Introduction

Platelets are critical regulators of thrombosis and inflammation [1]. Upon activation, platelets release prothrombotic (e.g., adenosine diphosphate (ADP)) or proinflammatory (e.g., cytokines or chemokines) compounds that promote endothelial activation and vascular lesions (thrombo-inflammation) [2]. Activated platelets augment vascular inflammation, resulting in atheroprogression [3]. Enhanced activity of circulating platelets is associated with an increase in cardiovascular events and death in patients with coronary artery disease [4]. Antiplatelet therapy is critical in preventing acute atherothrombotic events but does not control the progression of plaque and atherosclerotic disease.

Hyperlipidemia is associated with increased coagulation and platelet activity [5]. Recently, we showed that low-density lipoprotein (LDL) promotes platelet activation and platelet-dependent thrombus formation [6]. The interaction of oxidized low-density lipoprotein (OxLDL) via CD36 activates platelets and promotes thrombosis [7]. OxLDL and hyperlipidemia enhance the generation of Nox2-dependent reactive oxygen species (ROS) through CD36-PKC signaling and promote platelet hyperreactivity [8]. Thus, platelet hyperreactivity in hyperlipidemic patients may promote atheroprogression and contribute to acute atherothrombotic events in cardiovascular disease patients.

Proprotein convertase subtilisin/kexin type 9 (PCSK9) is critically involved in the regulation of plasma LDL levels [9]. PCSK9 belongs to the family of proprotein convertases and is synthetized as a soluble zymogen and released into the plasma compartment. PCSK9 is expressed in many tissues (e.g., liver, brain, kidney, and small intestine) and cell types, including endothelium, smooth muscle cells, and macrophages [10]. PCSK9 binds to the LDL receptor and reduces the uptake of LDL-particles from the extracellular compartment into cells, thereby increasing plasma LDL concentration. Thus, PCSK9 has a major impact on the maintenance of lipoprotein homeostasis. Inhibition of PCSK9 results in lowered plasma LDL and improves prognosis in patients with coronary artery disease [11].

Besides the control of cholesterol levels, pleiotropic effects of PCSK9 have been recently discussed [12,13]. Inhibition of PCSK9 may have effects beyond LDL metabolism. PCSK9 has been reported to be involved in inflammation, blood pressure regulation, adipogenesis, and glucose metabolism [14]. Furthermore, enhanced concentrations of PCSK9 have been found in cerebrospinal fluid in Alzheimer’s disease patients [15]. Recombinant PCSK9 modulates the function of macrophages and vascular inflammation [16]. PCSK9 has also been described as a modulator of platelet activation [17]. Recently, plasma PCSK9 has been shown to enhance platelet activation and in vivo thrombosis [18]. Inhibition of PCSK9 decreases platelet activation and ox-LDL metabolism [19].

Large clinical studies clearly show that antibody-based PCSK9 inhibition improves clinical outcomes in cardiovascular risk patients [11,20]. Furthermore, liver-specific targeting of PCSK9 mRNA with small interfering RNA achieves a significant reduction of LDL cholesterol in patients with atherosclerotic cardiovascular disease [21].

In the present study, we characterize the release of PCSK9 from platelets and its consequences for thrombo-inflammation. We show that platelets store and release PCSK9 upon activation and regulate aspects of thrombosis and inflammation beyond LDL metabolism.

## 2. Results

### 2.1. Platelet Count Is Associated with Plasma LDL Cholesterol

Hypercholesterinemia is associated with platelet hyperactivity [22]. In the present study, we evaluated platelet function in a consecutive cohort of patients (*n* = 707) with symptomatic coronary artery disease (Supplemental Table S1). A total of 48.5% of patients presented with chronic coronary syndrome (CCS) and 51.5% with acute coronary syndrome (ACS). We found that platelet count correlates with plasma LDL (*p* < 0.0001, *r* = 0.1541) and total cholesterol (*p* = 0.0001, *r* = 0.1430), but not with plasma HDL (*p* = 0.3350, *r* = 0.03647) or plasma triglycerides (*p* = 0.6008, *r* = −0.01981) (Supplemental Figure S1A). Platelet count was an independent predictor for LDL plasma levels documented through a univariate analysis of covariance (age, body mass index, gender, hypertension, diabetes, smoking, use of statins, aspirin, or P_2_Y_12_ inhibitor) (Supplemental Table S2). This implies that platelets contribute to the regulation of plasma cholesterol metabolism. Next, we asked whether residual platelet aggregation determined ex vivo is associated with plasma LDL. We found that TRAP (32 µM) induced ex vivo platelet aggregation correlates with LDL-plasma values (*p* = 0.0005, *r* = 0.1493) (Supplemental Figure S1B,C). This was not observed when ADP (6.5 µM), AA (484 µM), or collagen (3.2 mg/mL) were used as agonists for ex vivo platelet aggregation (Supplemental Figure S1B,C). This suggests that plasma LDL is associated with enhanced ex vivo platelet hyperreactivity.

### 2.2. Platelets Store and Release PCSK9 upon Activation

**Figure 1:**
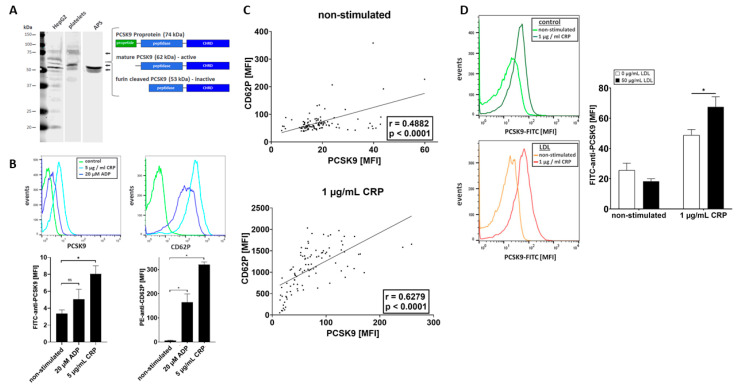
Release of PCSK9 from activated platelets. (**A**) Representative immunoblot images of PCSK9 expression in HepG2 cells, human platelets, and APS (CRP-stimulated, 5 µg/mL) from three independent experiments. (**B**) Platelet degranulation and release of PCSK9 was verified by surface expression of P-selectin (CD62P) using fluorochrome-conjugated PE-anti-CD62P and FITC-anti-PCSK9 antibodies by flow cytometry. Representative immunohistograms of three independent experiments are shown. PCSK9 signal was not significantly increased after ADP stimulation compared to non-stimulated platelets (*n* = 3), but CRP stimulation resulted in a statistically significant increase in anti-PCSK9-FITC binding (plotted: mean ± SEM; *n* = 4, Mann–Whitney test, ns = not significant, * *p* < 0.05). (**C**) Correlation of platelet activation and PCSK9 expression. Surface expression of CD62P and of PCSK9 on non-stimulated (upper panel) or CRP-stimulated platelets (1 μg/mL) (lower panel) were determined by flow cytometry. Data indicate Pearson’s correlation coefficient. (**D**) Effect of LDL on surface expression of PCSK9 on platelets. PRP was incubated with or without 50 µg/mL LDL and thereafter stimulated with CRP (1 µg/mL) or left unstimulated and stained with PCSK9-FITC and analyzed by flow cytometry. Left panel: representative immunofluorescence histograms of three independent experiments are shown. Right panel: LDL treatment had a significant effect on the PCSK9 expression on CRP stimulated platelets (plotted: mean ± SEM, *n* = 6, Mann–Whitney test, * *p* < 0.05).

Platelets store and release a variety of prothrombotic and proinflammatory compounds that are important regulators of thrombo-inflammation and atheroprogression [1]. Recently, it has been suggested that megakaryocytes and a subgroup of platelets express PCSK9. However, the role of platelet-derived PCSK9 in thrombosis and thrombo-inflammation remains poorly understood [12]. Thus, we explored whether platelets express and release PCSK9 upon activation. Human platelets were isolated to a high purity as previously described [23,24] and analyzed by immunoblotting using a specific anti-PCSK9 monoclonal antibody (mAb). We found that platelets express two PCSK9-immunoreactive bands with molecular mobility of 62 and 53 kDa (Figure 1A). In cultivated human liver cells (HepG2) as well as in platelets, an additional PCSK9 immunoreactive band was detected with 74 kDa, which represents the inactive PCSK9 proprotein (Figure 1A). This indicates that PCSK9 is present in significant amounts in human platelets.

Next, we asked whether PCSK9 is released upon platelet activation. Human platelets were isolated and stimulated with agonists such as ADP (20 µmol/L) and collagen related peptide (CRP, 5 µg/mL) [25]. Platelet degranulation was verified by surface expression of P-selectin (CD62P) using fluorochrome-conjugated PE-anti-CD62P and FITC-anti-PCSK9 antibodies by flow cytometry. We found that CRP (5 µg/mL) significantly enhanced surface expression of PCSK9 (*p* < 0.05) compared to non-stimulated platelets (Figure 1B). The release of PCSK9 in cleaved form was verified by immunoblotting (Figure 1A). We found that PCSK9 was present in the releasate in concentrations of 500 pg/mL per 300,000 plt/µL (data not shown). The results of our in vitro analysis were validated in a subgroup of patients (*n* ≥ 100) with coronary artery disease. Platelet PCSK9 correlated with CD62P surface expression both on non-activated and ex vivo CRP-stimulated platelets (1 µg/mL) (non-stimulated *p* < 0.0001, *r* = 0.4882; CRP-stimulated *p* < 0.0001, *r* = 0.6279) (Figure 1C).

**Figure 2:**
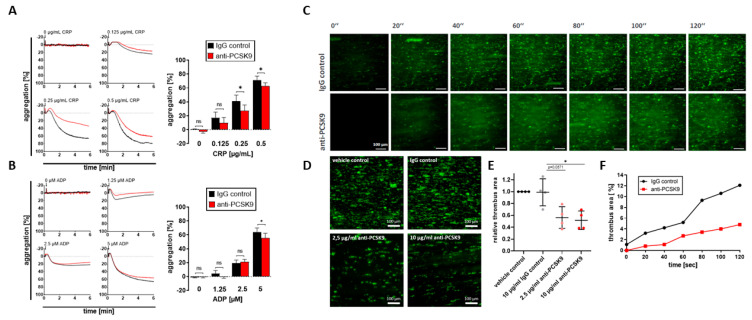
(**A**) Inhibition of PCSK9 decreased platelet aggregation. PRP was incubated with or without 15 µg/mL anti-PCSK9 (evolocumab). The samples were stimulated with CRP (0.125 µg/mL, 0.25 µg/mL, 0.5 µg/mL) or ADP (1.25 µM, 2.5 µM, 5 µM) and platelet aggregation was measured by light transmission aggregometry. Representative CRP-induced platelet aggregation curves demonstrating the effect of anti-PCSK9 treatment on platelet aggregation. Statistical analysis revealed the percentage of aggregation was reduced by anti-PCSK9 treatment (plotted: mean ± SEM; *n* ≥ 3, Wilcoxon signed-rank test, ns = not significant, * *p* < 0.05). (**B**) Representative ADP-induced platelet aggregation curves demonstrating the effect of anti-PCSK9 treatment on platelet aggregation. Statistical analysis revealed that the percentage of aggregation was reduced by anti-PCSK9 treatment under stimulation with 5 µM ADP (plotted: mean ± SEM; *n* ≥ 3, Wilcoxon signed-rank test, ns = not significant, * *p* < 0.05). (**C**) Anti-PCSK9 reduced platelet-dependent thrombus formation. Human whole blood was perfused over a collagen-coated surface (100 µg/mL) at a shear rate of 1000 s^−1^. Representative fluorescence images of thrombus formation over time for *n* ≥ 3 independent experiments. (Scale bar = 100 µm.) (**D**) Representative end-point fluorescence images for *n* ≥ 3 independent experiments. (Scale bar = 100 µm.) (**E**) Statistical analysis of thrombus coverage showed a significant decrease of the thrombus area in whole blood samples treated with anti-PCSK9 antibodies compared to the IgG control and the vehicle control (plotted: mean ± SEM; *n* ≥ 3, Mann–Whitney test, * *p* < 0.05). (**F**) Thrombus formation over 120 s, measured in one representative experiment.

To further analyze whether LDL modulates CD62P degranulation [6] and the release of PCSK9, isolated platelets were incubated with LDL (50 µg/mL) or control and activated with CRP (1 µg/mL). We found that PCSK9 release of platelets pretreated with LDL was significantly enhanced upon activation (Figure 2D) (*p* < 0.05), indicating that LDL propagates the release of platelet PCSK9.

### 2.3. Platelet-Derived PCSK9 Promotes Platelet Aggregation and Thrombus Formation under Flow Dynamic Conditions

Recently, a direct correlation of PCSK9 plasma levels and hyperreactivity of platelets in patients with acute coronary syndrome has been described [17]. In addition, recombinant PCSK9 potentiates platelet activation, as shown in in vitro experiments [17]. To explore whether platelet-derived PCSK9 modulates platelet function, we tested the effect of blocking anti-PCSK9 monoclonal antibodies on platelet aggregation. In the presence of anti-PCSK9 antibodies, ADP- and CRP-induced platelet aggregation were significantly attenuated, indicating that the release of PCSK9 in response to activation promotes platelet aggregation (Figure 2A,B). Furthermore, platelet-dependent thrombus formation on immobilized collagen was analyzed in the presence of blocking anti-PCSK9 (shear rate 1000 s^−1^). Compared to IgG-control experiments, anti-PCSK9 significantly reduced platelet-dependent thrombus formation (*p* < 0.05) (Figure 2C–F). Thus, the release of platelet-derived PCSK9 is a prominent prothrombotic factor that augments thrombus formation under flow.

### 2.4. Platelet-Derived PCSK9 Stimulates Migration of Monocytes

**Figure 3:**
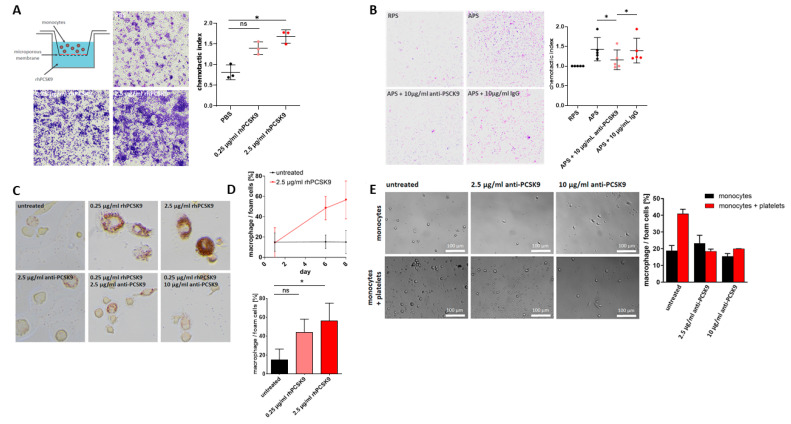
PCSK9 induced platelet migration. (**A**) Schematic view of experimental set up and representative images for three independent experiments. A significant chemotactic effect of rhPCSK9 (0.25 µg/mL and 2.5 µg/mL) on platelets could be observed (plotted: mean ± SEM; *n* = 3, Kruskal–Wallis test, * *p* < 0.05). (**B**) Representative images of the chemotactic effect of APS compared to resting platelet supernatants (RPS). This effect is prevented by anti-PCSK9 (10 µg/mL) but not by IgG control (plotted: mean ± SEM; *n* = 5, Mann–Whitney test, * *p* < 0.05). (**C**) Platelets propagate monocyte-derived macrophage development via PCSK9. Representative images of macrophage development under stimulation with rhPCSK9 (0.25 µg/mL, 2.5 µg/mL). The effect was inhibited by anti-PCSK9 antibodies (2.5 µg/mL, 10 µg/mL). (**D**) Macrophage/foam cells development over eight days under rhPCSK treatment (2.5 µg/mL). Statistical analysis of macrophage/foam cells development after eight days treatment with rhPCSK9 (0.25 µg/mL, 2.5 µg/mL) showed significant increase in macrophage/foam cells development (plotted: mean ± SEM; *n* ≥ 3, biological replicates; Kruskal–Wallis test, * *p* < 0.05). (**E**) Representative images of monocytes and monocytes/platelet co-culture. The analysis of macrophage development in monocyte/platelet co-culture after nine days treatment with anti-PCSK9 antibodies (2.5 µg/mL, 10 µg/mL) showed inhibition of macrophage development (plotted: mean ± SEM, *n* = 2).

Recent studies show that PCSK9 exerts pleiotropic effects beyond regulating the function of the LDL receptor [12]. Experimental mouse studies indicate that PCSK9 increases macrophage infiltration in atherosclerotic lesions [16]. To evaluate whether platelet-derived PCSK9 is functional and modulates migration of monocytes, we tested the effect of recombinant human PCSK9 (rhPCSK9) and of supernatant derived from activated platelets (APS) on monocyte migration using a modified Boyden chamber (Figure 3A). RhPCSK9 significantly stimulated monocyte migration, which could be attenuated in the presence of neutralizing anti-PCSK9 mAb (Figure 3B). This indicates that PCSK9 released from activated platelets modulates monocyte chemotaxis.

### 2.5. Platelet-Derived PCSK9 Promotes Differentiation of Monocytes into Macrophages/Foam Cells

Activated platelets are phagocytosed by monocytes and modulate their function and differentiation into macrophages/foam cells [26,27,28,29]. To test whether platelet-derived PCSK9 promotes monocyte differentiation into macrophages/foam cells, platelets were co-cultured with monocytes, and the formation of macrophages/foam cells was analyzed as previously described [30]. Recombinant PCSK9 promotes macrophage development in a time-dependent manner in monocyte cultures within 8 days, which could be attenuated in the presence of neutralizing anti-PCSK9 mAb (Figure 3C,D). Similarly, in platelet/monocyte co-culture experiments, macrophage development was significantly enhanced; in the presence of anti-PCSK9 mAb, it was significantly decreased (Figure 3E). This indicates that platelet-derived PCSK9 is highly involved in the platelet-dependent differentiation of monocytes into macrophages. 

### 2.6. Expression of PCSK9 in Platelet/Macrophage-Rich Areas in Atherosclerotic Carotid Tissue

**Figure 4:**
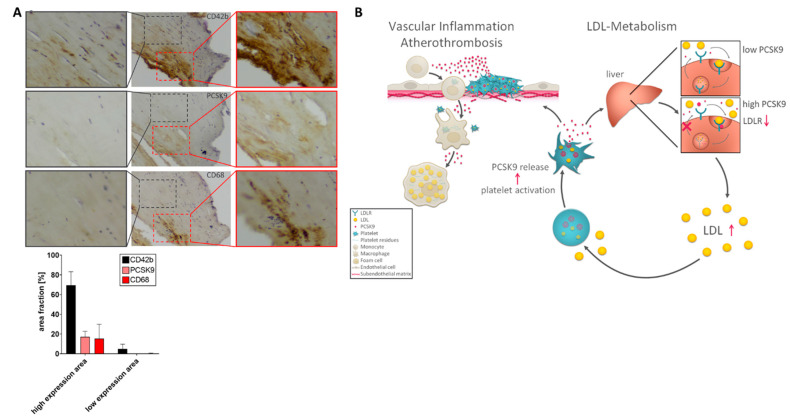
(**A**) CD42b, PCSK9, and CD68 immunostaining of human atherosclerotic carotid tissue. The representative immunostaining photomicrographs of CD42b, PCSK9, and CD68 expression in atherosclerotic tissue derived from endarterectomy specimen from carotid arteries are shown (*n* = 3). (**B**) Proposed role of platelet-derived PCSK9 in LDL metabolism and atherothrombosis. Enhanced release of PCSK9 from platelets promotes LDLR degradation and the increase of plasma LDL. Platelet-PCSK9 induces monocyte migration, macrophage development, and enhanced thrombosis.

To analyze whether PCSK9 is expressed in atherosclerotic tissue, endarterectomy specimens from diseased carotid arteries (*n* = 3) were immunostained. We found significantly enhanced PCSK9 immunostaining activity in diseased carotid tissue (Figure 4A). PCSK9 activity was primarily or exclusively found in areas of enhanced platelet immunoreactivity (CD42b), implying that platelets contribute substantially to PCSK9 expression in atherosclerotic tissue. Interestingly, in PCSK9-and platelet-positive areas, high immunoreactivity was found for macrophages (CD68) (Figure 4A).

## 3. Discussion

The major findings of the present study are the following. (i) Platelet count correlates with plasma-LDL cholesterol. (ii) Platelets store and release PCSK9 (pltPCSK9) upon activation, which is enhanced in the presence of LDL. (iii) PltPCSK9 promotes platelet aggregation and thrombus formation under flow dynamic conditions. (iv) PltPCSK9 is chemotactic for monocytes. (v) PltPCSK9 promotes differentiation of monocytes into macrophages/foam cells. (vi) PltPCSK9 is found in substantial amounts in atherosclerotic tissue. These findings indicate that circulating platelets may contribute to plasma levels of PCSK9. PltPCSK9 may contribute significantly to LDL metabolism in patients with coronary artery disease. Release of pltPCSK9 might be involved in the regulation of LDL plasma levels and may have an impact on thrombosis and vascular inflammation, and thus atheroprogression (Figure 4B). Therefore, it is tempting to speculate that the herein-described effects of pltPCSK9 on thrombosis and thrombo-inflammation might contribute to atherothrombosis. Beyond lowering LDL, PCSK9 inhibitors may also control thrombo-inflammation, a pleiotropic effect that contributes to risk reduction in patients with uncontrolled hypercholesterolemia and coronary artery disease.

Recently, inhibition of PCSK9 has become an effective and safe strategy to treat patients with uncontrolled hyperlipidemia and coronary artery disease [31]. Administration of PCSK9 inhibitors significantly improved quality of life [32] and reduced adverse clinical events and mortality in CAD patients [20,33]. PCSK9 belongs to the family of proprotein convertases that activate various proteins. PCSK9 is abundantly expressed in many tissues and cell types. PCSK9 binds to the LDL receptor (LDLR) and triggers endocytosis of LDL from the extracellular compartment into primarily liver cells, thus increasing LDL plasma levels. Blocking PCSK9 results in the recycling of LDLR and promotes further clearance of plasma LDL. Plasma levels of PCSK9 correlate with LDL. The association between plasma PCSK9 and LDL can be significantly modified through lipid-lowering drugs [31]. Furthermore, plasma PCSK9 levels correlate with platelet count in patients with CAD, implying a pathophysiological link between PCSK9 and the platelets system [34]. In the present study, we found that platelets are a source of PCSK9, which is released from activated platelets and may contribute to the PCSK9 plasma pool. Since enhanced platelet activation and secretion—especially in hypercholesterolemia—is well documented in patients with CAD, we hypothesized that platelets contribute to PCSK9 activity and thus the regulation of LDL plasma levels. Plasma levels of PCSK9 have been associated with CAD; however, the results are conflicting [35,36,37,38,39]. Recent results of the GLAGOV clinical trial suggest that the PCSK9 inhibitor evolocumab reduces the progression of atheroma volume in patients with CAD [40].

Previously, we showed that LDL augments platelet activation in vitro and in vivo [6]. Our present data provide evidence that LDL also promotes PCSK9 release from activated platelets. Thus, we hypothesized that the release of pltPCSK9 triggers LDL plasma levels, which in turn further enhance the release of pltPCSK9 and systemic plasma PCSK9 concentrations (Figure 4B). Thus, we postulate that platelets are a hitherto unrecognized compartment, which is (patho)physiologically relevant in lipid metabolism.

Circulating PCSK9 and platelet hyperreactivity have been shown to be associated with carotid intima media wall thickness and coronary plaque inflammation, suggesting a role of PCSK9 in vascular inflammation and atheroprogression [33,34,41]. In the present study, we found that PCSK9 is found in significant amounts in human atherosclerotic tissue, primarily or exclusively in areas with enhanced platelet and macrophage immunoreactivity. This confirms previous studies that PCSK9 accumulates in atherosclerotic tissue. Ferri et al. showed that PCSK9 found in atherosclerotic tissue is derived from smooth muscle cells (SMCs) [42]. Our data show that platelets are an additional source of PCSK9 within diseased arteries in addition to SMCs. Our in vitro experiments provide evidence that pltPCSK9 stimulates the migration of monocytes and fosters macrophage and foam cell development. This effect can be substantially reduced by inhibiting PCSK9. This indicates that PCSK9 is a chemotactic and proinflammatory factor that promotes atherosclerosis. Furthermore, we found that platelet-derived PCSK9 enhances platelet-dependent thrombus formation, which can be decreased by PCSK9 inhibition. Thus, our data establish pltPCSK9 as a relevant factor for atherothrombosis and thrombo-inflammation, which might at least partially be relevant for the highly efficient effects of PCSK9 inhibitors to reduce mortality and other adverse cardiovascular events in coronary artery disease.

Besides its critical role in the regulation of LDL metabolism, there is increasing evidence that PCSK9 has multiple pleiotropic effects [43]. This study and previous ones indicate that PCSK9 is involved in the pathophysiology of atherosclerosis and thrombosis. Furthermore, PCSK9 is involved in glucose metabolism, renal function, immune responses, inflammation, and sepsis. Whether these PCSK9 effects are of clinical significance remains to be shown. At present, we do not provide direct evidence how and whether platelets affect hepatic lipid metabolism. Previously, we showed in mice that platelets accumulate in the liver microcirculation during the development of non-alcoholic fatty liver disease [24]. Thus, enhanced activation of platelets within the liver circulation may contribute to enhanced PCSK9 concentration in the hepatic cell milieu and therefore may have an impact on LDL recycling. One may speculate that modulation of platelet activation has a favorable effect on atheroprogression and liver inflammation. However, at present, this is speculative and requires solid experimental data.

## 4. Materials and Methods

### 4.1. Study Patients

We included 707 consecutive patients with symptomatic CAD (Supplemental Table S1). The study was approved by the local ethics committee (141/2018B02, 240/2018B02), and all patients gave written informed consent. The experiments were performed in accordance with the ethical standards laid down in the Declaration of Helsinki.

### 4.2. Chemicals and Reagents

Recombinant human PCSK9, mouse monoclonal anti-PCSK9, and polyclonal goat IgG were from R&D Systems/BioTechne, Minneapolis, MN, USA. Anti-human CD62P-PE (clone AK4) and anti-human CD42b-APC (clone HIP-1) were from Biolegend (San Diego, CA, USA). LDL was purchased from Kalen Biomedical (Germantown, MD, USA). Mouse control IgG was procured from Santa Cruz Biotechnologies (La Jolla, CA, USA). Repatha (evolocumab) was from Amgen (Thousand Oaks, CA, USA) and conjugated with fluorescein isothiocyanate (FITC, Sigma-Aldrich/Merck, Darmstadt, Germany) by standard methods.

### 4.3. Isolation of Peripheral Blood Platelets and Monocytes and Platelet Flow Cytometry

Platelets and monocytes were isolated according to established protocol as described [23]. Platelet-rich plasma (PRP) flow cytometry was performed according to standard methods [23].

### 4.4. Immunoblotting

Western blot analysis was performed as described previously [44]. PCSK9 was detected using a primary anti-PCSK9 antibody (polyclonal goat IgG, R&D Systems/BioTechne, Minneapolis, MN, USA) and an IR-Dye 680 RD donkey anti-goat IgG as secondary antibody (LI-COR, Lincoln, NE, USA). 

### 4.5. In Vitro Monocyte Migration Assay

Monocyte migration was analyzed in a modified 48-well Boyden chamber (Neuro Probe Inc., Gaithersburg, MA, USA) as described [23]. Monocytes were added in the upper compartment, and recombinant human PCSK9, evolocumab, supernatant obtained from resting (RPS), or activated platelets (APS) were added to the lower compartment in concentrations as indicated. After four hours, monocytes associated with the filter membrane were detected by May–Grünwald/Giemsa staining (Merck Millipore, Darmstadt, Germany). Migrated monocytes on the filter were detected using a microscope (Nikon Eclipse Ti2-A, Nikon, Tokyo, Japan).

### 4.6. Platelet Aggregometry

In clinical samples, platelet aggregation was assessed by impedance platelet aggregometry according to standard procedures (Multiplate Analyzer, F. Hoffmann-La Roche Ltd., Basel, Switzerland). As agonists, thrombin-receptor-activating peptide (TRAP) 32 µM, ADP 6.5 µM, arachidonic acid (AA) 484 µM, and collagen 3.2 mg/mL were used [45]. Additionally, light transmission aggregometry was performed for ex vivo experiments according to the standard protocol [46].

### 4.7. Ex Vivo Thrombus Formation-Flow Chamber Assay

Whole blood was perfused over collagen-coated (100 µg/mL) cover slips with a shear rate of 1000 s^−1^ and photo-documented after the blood perfusion was stopped [23]. The thrombus area was quantified in photo-documented images (Nikon Eclipse Ti2-A, NIS-Elements AR, Nikon, Tokyo, Japan).

### 4.8. Monocyte Differentiation into Macrophages and Foam Cells

Monocytes and platelets were co-cultured in RPMI-1640 medium for eight days as previously described [26] in the presence or absence of rhPCSK9 and anti-PCSK9 mAb as indicated. Differentiated macrophages/foam cells were characterized by an increase in cell diameter (>10 µm) and were quantified using post-acquisition image analysis using ImageJ software (National Institutes of Health, Bethesda, MD, USA) as described previously. 

### 4.9. Immunostaining of Atherosclerotic Carotid Samples

Paraffin tissue samples were obtained from a carotid endarterectomy specimen. Immunohistochemical staining for PCSK9 (anti-m/rPC9 affinity-purified goat IgG, AF3985, R&D Systems/BioTechne, Minneapolis, MN, USA), CD68 (anti-human mouse monoclonal IgG F1118, Santa Cruz Biotechnologies, Dallas, TX, USA), and CD42b (anti-human mouse monoclonal IgG B0712, Santa Cruz Biotechnologies, Dallas, TX, USA) were then undertaken using HRP-DAB staining systems (anti-goat kit, CTS008, R&DSystems/BioTechne, Minneapolis, MN, USA, anti-mouse kit, CTS0002, R&D Systems/BioTechne, Minneapolis, MN, USA) according to the manufacturer’s protocol.

### 4.10. Statistics

Graphs were created with the GraphPad Prism software (GraphPad Software, Inc., La Jolla, CA, USA) and represent biological replicates as indicated in the figure legends. Data are presented as dot plots with mean ± S.E.M. All data were tested for significance using GraphPad Prism software (GraphPad Software, Inc., La Jolla, CA, USA), setting statistical significance at *p* < 0.05 for an unpaired t-test to compare two sets of data with normal distribution. Data of non-normal distribution were tested using the Mann–Whitney U test at 95% CI. Patient data were analyzed using SPSS version 26.0 (SPSS Inc., Chicago, IL, USA). Correlations were assessed by Pearson’s rank correlation coefficient (*r*). General linear models were established using univariate analysis of covariates to show independent associations of platelet count with plasma LDL cholesterol. Variables entered into the model included age, BMI, sex, arterial hypertension, diabetes mellitus type II, smoking, statins, and ASA.

## 5. Limitations

Our current data do not provide direct evidence of the role of platelets or pltPCSK9 on the regulation of plasma LDL levels. We are fully aware that our clinical data as presented are descriptive. Although it is tempting to speculate that the results of our in vitro data may reflect aspects of the in vivo situation, conclusive proof is missing so far.

## Data Availability

The data presented in this study are available in this paper.

## Supplementary Materials

The following are available online at https://www.mdpi.com/article/10.3390/ijms222011179/s1.
