# Supplementary material for: Platelet-Derived PCSK9 Is Associated with LDL Metabolism and Modulates Atherothrombotic Mechanisms in Coronary Artery Disease
Source: Int J Mol Sci. 2021 Oct 16;22(20):11179. doi: 10.3390/ijms222011179 (PMC8538687; doi:10.3390/ijms222011179)
Supplement: Supplementary file 1 [file ijms-22-11179-s001.zip › ijms-1384776-supplementary.pdf]

Supplementary Materials for

Platelet-Derived PCSK9 is Associated with LDL Metabolism and Modulates Atherothrombotic Mechanisms in Coronary Artery Disease

Álvaro Petersen-Uribe, Marcel Kremser, Anne-Katrin Rohlfing, Tatsiana Castor, Kyra Kolb, Valerie Dicenta, Frederic Emschermann, Bo Li, Oliver Borst, Dominik Rath, Karin Anne Lydia Müller and Meinrad Paul Gawaz*

Department of Cardiology and Angiology, University Hospital Tübingen, Eberhard Karls University Tübingen, Tübingen 72076, Germany; Alvaro.Petersen@med.uni-tuebingen.de (A.P.-U.); Marcel.Kremser@med.uni-tuebingen.de (M.K.); Anne-Katrin.Rohlfing@med.uni-tuebingen.de (A.-K.R.); Tatsiana.Castor@med.uni-tuebingen.de (T.C.); Kyra.Kolb@med.uni-tuebingen.de (K.K.); Valerie.Dicenta@med.uni-tuebingen.de (V.D.); Frederic.Emschermann@med.uni-tuebingen.de (F.E.); Bo.Li@med.uni-tuebingen.de (B.L.); Oliver.Borst@med.uni-tuebingen.de (O.B.); Dominik.Rath@med.uni-tuebingen.de (D.R.); K.Mueller@med.uni-tuebingen.de (K.A.L.M.)

* Correspondence: Meinrad.Gawaz@med.uni-tuebingen.de; Tel.: +49-70712983688

This PDF file includes:

Tables S1 to S2
Figures S1

Supplemental Table S1: Baseline characteristics of the patients

	**All (n=707)**
Age, years, mean (±SD)	70 (±12)
Male, n (%)	500 (70.7)
Body mass index, mean (±SD)	27.5 (±5.1)
**Cardiovascular risk factors, n (%)**	
Arterial hypertension	630 (89.1)
Hyperlipidemia	459 (64.9)
Diabetes mellitus	229 (32.4)
Current smokers	126 (17.8)
Ex-smoker>6mo	141 (19.9)
Obesity	237 (33.5)
Atrial fibrillation	167 (23.6)
Prior CABG	39 (5.5)
Prior MI	153 (21.6)
Chronic kidney disease	88 (12.4)
**Disease, n (%)**	
ACS	365 (51.6)
NSTEMI	194 (27.4)
STEMI	63 (8.9)
Unstable angina	108 (15.3)
CCS	342 (48.5)
**Transthoracic Echocardiography**	
Ejection fraction, %, mean (±SD)	53 (±10)
**Medication at blood sampling, n (%)**	
Oral anticoagulation	155 (21.9)
ACEi/ARB	595 (84.2)
Aldosterone inhibitors	160 (22.6)
Diuretics	263 (37.2)
Calcium chanel blockers	250 (35.4)
Beta blockers	489 (69.2)
Statins	586 (82.9)
ASA	503 (71.1)
Clopidogrel	263 (37.2)
Ticagrelor	160 (22.6)
Prasugrel	82 (11.6)
**Laboratory parameters**	
Leucocytes, 1000/µl, mean (±SD)	8.4 (±2.9)
Platelets, 1000/µl, mean (±SD)	226 (±69)
Creatinin, mg/dl, mean (±SD)	1.2 (±2.2)
Total cholesterol, mg/dl, mean (±SD)	165 (±45)
LDL-cholesterol, mg/dl, mean (±SD)	103 (±42)
HDL-cholesterol, mg/dl, mean (±SD)	47 (±20)
Triglycerides, mg/dl, mean (±SD)	148 (±105)
C-reactive protein, mg/dl, mean (±SD)	1.2 (±3.0)
HbA1c ,% , mean (±SD)	6.3 (±1.0)

ACEi = angiotensin-converting enzyme inhibitors; ACS = acute coronary syndrome; ARB = an-giotensin II receptor blockers; ASA = acetylsalicylic acid; CABG = coronary artery bypass; CCS = chronic coronary syndrome; HbA1c = glycated haemoglobin;MI = myocardial infarction; NSTEMI = Non-ST-elevation myocardial infarction; STEMI = ST-Elevation Myocardial Infarction.

Supplemental Table S2: Univariate Analysis of Covariance

	p value	Confidence Interval
Platelets, 1000/µl	**0.01**	(0.01 – 0.11)
Age, years	**<0.01**	(-0.87 – -0.24)
Body mass index	0.99	(-0.64 – 0.64)
Gender	**0.01**	(2.8 – 17.1)
Arterial hypertension	0.34	(-15.9 – 5.5)
Diabetes mellitus	**0.01**	(-15.9 – -2.4)
Current smokers	0.51	(-12.2 – 6.1)
Statins	**<0.01**	(-34.0 – -16.3)
Acetylsalicylic acid	**<0.01**	(3.5 – 17.8)
P_2Y_{12} inhibitor	0.275	(-3.431 – 12.038)

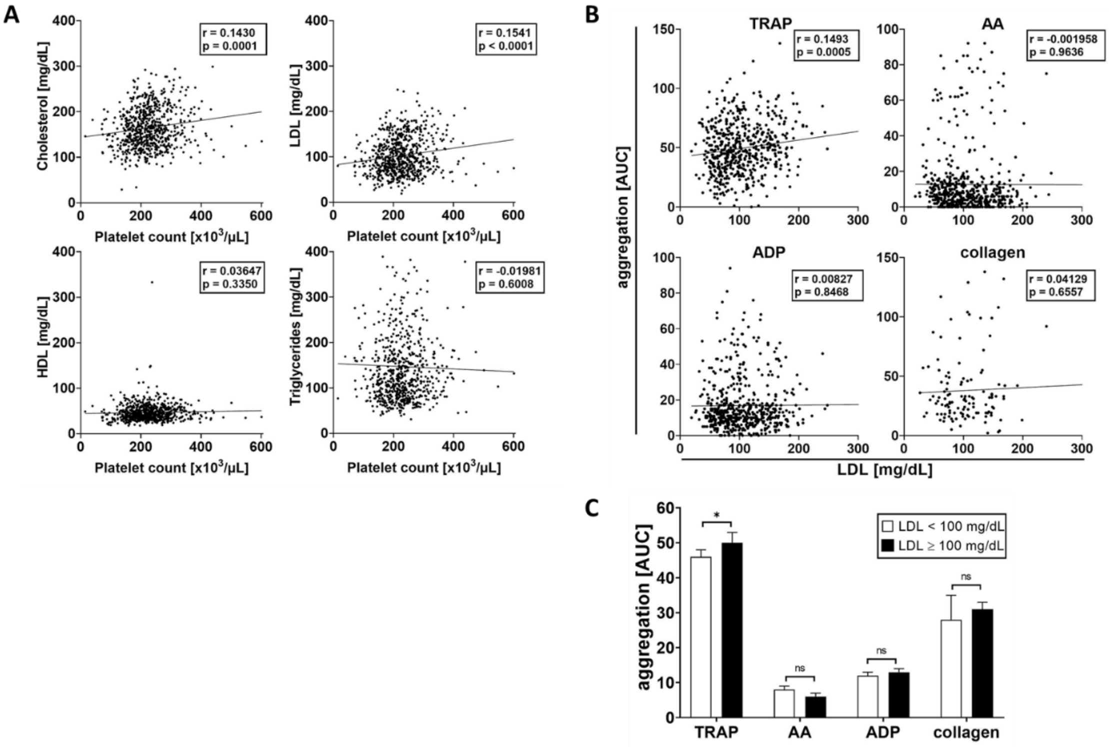

Supplemental Figure S1. Correlation of platelet count and plasma lipoproteins in symptomatic coronary artery disease (CAD). Data indicate Pearson's correlation coefficient (A). (B) Correlation of platelet reactivity and LDL plasma levels. The relationship between plasma LDL and platelet aggregation in whole blood was measured by impedance aggregometry in a subgroup of patients with symptomatic CAD (n≥500). Platelets were exposed to different activators like thrombin receptor activating peptide (TRAP, 32 μM) (n=545), arachidonic acid (AA, 484 μM) (n=545), adenosine diphosphate (ADP, 6.5 μM) (n=548) or collagen (3.2 mg/mL) (n=119). Results are presented as area under the curve (AUC) in units (U). Data indicate Pearson's correlation coefficient (B), (C) depicts mean ± SEM of aggregation.
